# A statistical analysis of the three-fold evolution of genomic compression through frame overlaps in prokaryotes

**DOI:** 10.1186/1745-6150-2-22

**Published:** 2007-09-18

**Authors:** Fabrizio Lillo, David C Krakauer

**Affiliations:** 1 Santa Fe Institute, 1399 Hyde Park Road, Santa Fe, NM 87501, USA; 2 Dipartimento di Fisicae Tecnologie Relative, Università di Palermo, Viale delle Scienze, I-90128 Palermo, Italy

## Abstract

**Background:**

Among microbial genomes, genetic information is frequently compressed, exploiting redundancies in the genetic code in order to store information in overlapping genes. We investigate the length, phase and orientation properties of overlap in 58 prokaryotic species evaluating neutral and selective mechanisms of evolution.

**Results:**

Using a variety of statistical null models we find patterns of compressive coding that can not be explained purely in terms of the selective processes favoring genome minimization or translational coupling. The distribution of overlap lengths follows a fat-tailed distribution, in which a significant proportion of overlaps are in excess of 100 base pairs in length. The phase of overlap – pairing of codon positions in complementary reading frames – is strongly predicted by the translation orientation of each gene. We find that as overlapping genes become longer, they have a tendency to alternate among alternative overlap phases. Some phases seem to reflect codon pairings reducing the probability of non-synonymous substitution. We analyze the lineage-dependent features of overlapping genes by tracing a number of different continuous characters through the prokaryotic phylogeny using squared-change parsimony and observe both clade-specific and species-specific patterns.

**Conclusion:**

Overlapping reading frames preserve in their structure, features relating to mutational origination of new genes, but have undergone modification for both immediate benefits and for variational buffering and amplification. Genomes come under a variety of different mutational and selectional pressures, and the structure of redundancies in overlapping genes can be used to detect these pressures. No single mechanism is able to account for all the variability observed among the set of prokaryotic overlapping genes but a three-fold analysis of evolutionary events provides a more integrative framework.

**Reviewers:**

This article was reviewed by Eugene Koonin, Marten Huynem, and Han Liang.

## Background

One of the remarkable discoveries arising from the study of microbial genomes is that a single sequence of nucleic acid bases can encode multiple different genes in overlapping reading frames [1-5]. This represents compression of genetic information, much like the compression of sound or video data in MP3 or MPEG formats [6]. Compression is possible when there are redundancies in a message, and genomic compression minimizes redundancies in a nucleotide sequence by exploiting degeneracies in the assignment rules of the canonical genetic code. A common framework according to which we might understand overlapping genes has not been available, and recent papers have called for some form of unified, evolutionary explanation [7]. In this paper, we analyze length, polarity and phase properties of genetic overlap in prokaryotes adopting a common framework of genome compression [8]. We consider three periods of the evolutionary process starting with the origin of overlap and the subsequent direct and indirect selection pressures acting on overlap length and phase. To clarify the exposition we call these: (1) mutational origins, (2) immediate benefits, and (3) variational benefits.

The mutational origin of overlapping genes typically involves point mutations to stop codons. These lead to the extension of a reading frame over an existing gene producing a genetic *palimpsest *in which one gene is inscribed above another. Once an overlap has arisen it can either impose a cost in terms of increased mutational sensitivity and regulatory interference [9], it can have no effect and behave as a neutral trait, or confer benefits. Benefits can be either immediate or variational.

The immediate benefits of overlap are features that have a direct impact on the replicative or metabolic efficiency of a genome within a single generation. Immediate benefits are often hypothesized to account for variation in the length of the overlapping sequences. The two immediate benefits of overlap are: (1) that overlapping sequences evolve under a pressure to foster replicative compression thereby increasing the rate of DNA or RNA synthesis for a fixed number of genes, and/or (2) for regulatory compression, reducing the independent regulatory overheads on different genes. In viruses immediate benefits are considered a dominant pressure on genome size and multiple overlapping genes are frequently present, often with genes embedded within genes.

The variational benefits are features that influence the standing, phenotypic variation in the population and thereby modulate the strength of selection on a lineage. These features are intended to account for variation in the overlap phases. The two variational benefits are that: (1) variation in the overlap phase is chiefly to be accounted for in terms of a compensatory alignment of genes leading to a maximum mutational redundancy, or (2) its inverse – aligning overlapping genes in order to encourage greater sequence mutability by promoting multiple amino acid replacements upon a single nucleotide mutation.

All three sets of evolutionary process are tested against data derived from prokaryotic genomes. Previous work on overlap [2,10,11] has stressed sequence polarity and length variability, typically in relation to mutational origins and immediate benefits associated with translation or replication rates. These prior studies tend to emphasize the detailed properties of the overlapping genes in a small sample of species [12,13]. A summary of a few related earlier papers is given in Table 1. In this paper we explore the selective implications of statistical regularities in phase diversity, we explicitly introduce statistical, null models against which we compare the data, and we identify the ongoing legacy of mutational origins in established patterns of overlap in a large set of prokaryotic genes. A couple of observations that becomes apparent when reading over the many previous studies on this topic are (Table 1) an emphasis on gene regulation in eukaryotes and genome compression in prokaryotes, and (2) a clear division into those studies focused on the mutational origin of overlap favoring neutral hypotheses, and those focusing on ongoing selection pressures to minimize the mutational load of overlap while maximizing the coding potential. Throughout this paper it will be stressed that while these positions can serve as an useful focus for investigation, a full understanding of overlap requires an appreciation of all of these evolutionary processes and their corresponding evolutionary sequence.

**Table 1:** A few representative studies on overlapping genes, organized alphabetically with summary of principle conclusions and data type

**Authors**	**Year**	**Genomes**	**IE**	**VE**	**SM**	**Genes & Homology**	**Evolutionary Process**
Lillo & Krakauer	2007	58 Prokaryote	RC & TC	MA & MB	NS	Study of E. coli genome	Neutrality & Selection
Boi et al [2]	2004	? Eukaryote	GR	NS	NS	NS	Selection on regulation
Das & Yanofsky [3]	1989	1 Prokaryote	TC	NS	NS	Study of 2 genes	Selection on expression
Fukuda et al. [6]	1999	2 Prokaryote	NS	NS	NE	Study of 480 & 677 genes	Neutral mutation to stop codon
Fukuda et al [7]	2003	3 Prokaryote	NS	NS	NS	NS	Neutral mutation to stop and start
Johnson & Chisholm [13]	2004	198 Prokaryote	TC	NS	NS	Study of 2 genomes	Selection on expression
Krakauer [15]	2000	NS	RC & TC	MA	NS	NS	Selection on replication and expression
Krakauer [16]	2002	NS	RC & TC	MA & MB	NS	NS	Selection on replication and expression
Makalowska et al. [22]	2005	7 Eukaryote	GR	NS	NS	NS	Neutrality
Merino et al. [25]	1994	28 Prok & 8 Euk	GR	NS	NS	Study of E. coli genome	Selection on regulation
Miyata & Yasunaga [24]	1978	1 Prokaryote	NS	MA	NS	Study of -x174	Selection on replication
Normark et al. [26]	1983	? Prokaryote	TC	NS	NS	NS	Selection on expression
Oppenheim & Yanofsky [28]	1980	1 Prokaryote	TC	NS	NS	E. coli Trp operon	Selection on expression
Pavesi et al. [29]	1997	? Viral	NS	MB	IC	NS	Selection on coding
Rogozin et al. [30]	2002	? Prokaryotes	NS	MB	NS	NS	Selection on coding
Sakharkar et al [31]	2005	9 Prokaryotes	RC	NS	NS	Study of 2 genomes	Selection on replication
Sander & Schulz [32]	1979	2 Prokaryote	NS	MB	NS	Study of SV40 & -x174 genes	Selection on coding
Welch et al. [39]	2000	1 Prokaryote	TC	NS	NS	Study of V anguillarum	Selection for iron biosynthesis

Key: IE – Immediate Effect, VE – variational effect, SM – Statistical Model NS – Not in Study, TC – translational coupling, RC – replicative compression, GR antisense gene regulation MB – mutational buffering, MA – mutational amplification, NE – Neutral expectation under statistical model, IC – Shannon information content of overlap. The ? symbols records an unknown number lineages. The column labeled "Genes and detailed homology" captures the pattern of many studies to couple the statistics of large databases with the detailed analysis of a small number of overlapping genes from well studied genomes. The difference between selection on expression versus selection on regulation seeks to discriminate between co-expression (as in a codon) and active inhibition or activation (such as through anti-sense), The difference between selection on replication and selection on coding reflects a difference only in emphasis as both imply a greater number of genes in a shorter sequence.

## 1 Mutation, Length and Phase

In this section we introduce several evolutionary processes by briefly stepping through a series of events starting with a random mutation producing an overlap, followed by an immediate benefit of the overlap leading to the fixation the overlapping variant, and the subsequent differential selection acting on overlap phase to satisfy variational constraints.

### 1.1 Origination

The differences in genetic overlap characteristics that we observe among species frequently appears to be a legacy of their stochastic mechanism of origination [14,15,33]. In other words, overlap can arise by mutation and behave as if it were selectively neutral. One mechanism of origination is that the overlapping sequences arise through random mutations to contiguous nucleotides of two genes in a single strand, leading to the formation of a 4-base overlap to include a stop and start codon. Subsequent ribosomal frame shifting at the stop of the mRNA transcript would allow for the initiation of translation of a second gene. It has been suggested that for two *Mycloplasma *species this mechanism accounts for around 7% of cases of overlap [33].

### 1.2 Minimization

Once overlap is established in a single genome it must fix in a population. Fixation can be neutral or selective. Immediate selective benefits can arise through both more efficient regulation and increased rates of replication [8,9,11,17]. In replicative compression, the total genome length is minimized by increasing the number of overlapping genes without loss of protein function. All else being equal, overlap can lead to an increase in the rate of replication by reducing the number of bases that need to be synthesized. For a constant per base per genome mutation rate, reducing genome size also reduces the mutation load which is an important factor in promoting fixation of genomes reduced through deletion events [18]. Having said this, in some cases maximal growth rates become independent of genome size suggesting alternative rate limiting reactions [19]. Regulatory compression works in two ways, both of which eliminate the need for independent transcription of genes. One mechanism, which applies to overlapping genes in an operon, is co-translation. Co-translation renders the translation of a distal gene dependent on the prior translation of an upstream gene [20,2]. Co-translation can increase the rate of protein synthesis and ensures a strict stoichiometry among gene products. In the second mechanism, overlapping genes are alternately translated through infrequent ribosomal frame-shifts of a single mRNA transcript. Each frameshift favors one polycistronic gene over another [22].

### 1.3 Phase modification

One consequence of the triplet code, is that overlapping sequences can overlap in different translational phases – the incidence of codon position pairings between genes. For example, codon position 1 of gene 1 overlaps with codon position 3 of gene 2 [9,10]. The evolutionary stability of variation in overlap phase can not be explained simply in terms of increased translation or replication rates within individuals, as these are largely insensitive to phase. Phase is a "variational property" meaning that it does not contribute directly to organismal replication rates within one generation, but indirectly, by modulating the phenotypic effects of point mutations on descendant sequence variability. In addition to phase differences stemming from neutral mutations, we consider the variational principles of *redundancy *[23] and *amplification *[24] in explaining phase variation in overlapping genes.

Redundancy describes the ability of overlapping sequences to minimize non-synonymous substitutions at the level of two or more proteins encoded by a single overlapping sequence. Whereas overlap can increase rates of replication, it is also expected to increase mutation load by increasing the number of amino acid substitutions for a single base pair mutation. Overlapping sequences can compensate for this sensitivity by pairing degenerate codon positions with non-degenerate codon positions through a suitable choice of phase.

Amplification or increased "mutational effect", relates to increased protein polymorphism following mutations to genes occupying certain phases of overlap. Amplification is achieved by pairing non-degenerate codon positions of two or more overlapping sequences, thereby maximizing the probability of non-synonymous substitutions. Another way to think about overlap phase is that it behaves analogously to 'adaptive-mutation' in prokaryotes [25], whereby genes experience different effective rates of amino acid substitution by virtue of differential overlap phase [26].

### 1.4 A multi-step process

As the foregoing paragraphs have suggested, patterns of overlap are difficult to interpret, as at least three different mechanisms can be adduced for extant patterns of diversity: mutations leading to overlap with neutral effects and slow fixation rates, selective benefits accruing through replicative and regulatory minimization leading to rapid fixation, and indirect selective benefits associated with increased or reduced standing variation through changes in phase. In this paper we shall endeavor to tease apart these contributions, bearing in mind that they are all likely to play an enduring role.

## 2 Methods

**Figure 1:**
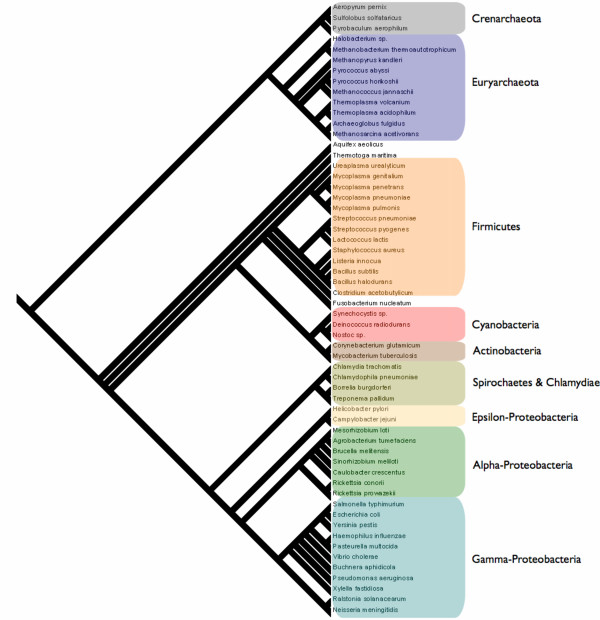
Supertree of 58 prokaryotic species with principal clades indicated. The tree is derived from a meta-analysis of prokaryotic trees constructed using complete genome sequences [29] and conserved indels [30]. The major clades are annotated.

We analyze 58 different species of prokaryotes whose genomes have been completely sequenced (See Figure 1) We selected the species (and the corresponding strains) that are included in the COG (Clusters of Orthologous Groups of proteins) database [27,28]. Each COG consists of individual proteins or groups of paralogs from at least 3 lineages and thus corresponds to an ancient conserved domain. We have analyzed all bacterial species available at the COG database except *Mycobacterium leprae*, because it is known that in this genome less than half of the genome contains functional genes and pseudogenes are abundant. For species, such as *E. coli*, for which more strains are annotated we selected only one of them to avoid duplicates. It is worth noting that by repeating the analysis for a larger set of 83 species and by including all the genes in the annotation, our results do not change significantly. While the use of the COGs database reduces the false positive rate in our analysis, we do not have access to the ribosomal binding sites for all overlapping genes, which would further increase our confidence.

In our dataset there are 13 Archaea (10 Euryarchaeota and 3 Crenarchaeota) and 45 Bacteria. The length of genomes range from 580 kbp to 7 Mbp. A scatter plot of the number of genes plotted against the genome length shows a good linear relation (*R*^2 ^= 0.98), with a density of one gene per 1130 bp. The proportion of noncoding material in the genome is around 13%, with extremal values of 6% (*Thermotoga maritima*) and 26% (*Methanosarcina acetivorans*). There is no evident difference in these two properties between Archaea and Bacteria.

In the following we consider only overlapping sequences between two (or more) genes that are both annotated as belonging to one COG. In this way we minimize the incidence of annotation errors. On average 76% (min = 58%, max = 99%) of the annotated genes are annotated as belonging to one COG. This makes our analysis representative of the behavior of genes in the genome. For increased confidence we have repeated our analysis for a more restrictive subset. We include in the restricted set only those overlapping sequences that are conserved in two or more species. A sequence is conserved in two genomes if in the two species the overlap is created by two pairs of genes belonging to the *same *COGs. We have found that our results are replicated when using this restricted data-set.

**Figure 2:**
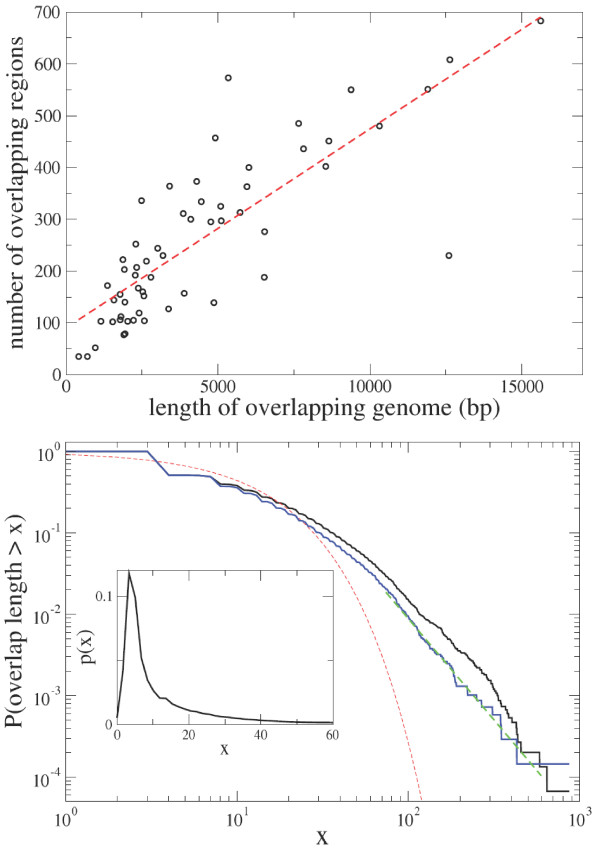
Top. Scatter plot of the number of overlapping sequences versus the total number of overlapping bases for the 58 investigated genomes. The line is a best linear fit and gives a typical overlap length of 26 bp. The analysis is performed by considering only overlapping genes which are both annotated in the COG database. Bottom. Cumulative density function of the length of overlapping sequences in the whole dataset (14, 958 overlaps). The red dashed line is the best fit of the distribution with an exponential function exp(-*ax*). The blue line is the cumulative density function only for conserved ORs (see text). The green dashed line is a best fit and the estimated exponent is 2.5. The plot is log-log. The inset shows the probability density function of overlap length in the range of short ORs.

An overlapping sequence is a portion of the genome that encodes some part of two or more protein coding genes. We do not include in our analysis pairs of genes in which one of the two genes is completely contained (embedded) within the other. The number of overlapping sequences is relatively high when compared to the total number of genes in the genome. The mean number of genes involved in at least one overlap is 27% with extremal values 8% (*Nostoc sp.*) and 56% (*Aquifex aeolicus *and *Thermotoga maritima*). As a consequence, in prokaryote genomes a significant proportion of genes have an overlapping sequence. Top panel of Figure 2 shows a scatter plot of the number of overlaps versus the total number of overlapping bases. A linear fit on the data gives an estimate of the mean length of the overlapping sequences equal to ≃26 bp (correlation coefficient *ρ *= 0.82, *p *= 10^-15^).

## 3 Results and Discussion

### 3.1 Length and Strand Statistics

We have investigated the length distributions of overlapping sequences. We include all overlapping sequences of all genomes conditioned on the constraint that both genes in the overlap are annotated in the COG database. The total number of overlaps are 14, 958. The bottom panel panel of Figure 2 illustrates the probability that an overlapping sequence is longer than *x *as a function of *x*. The plot is log-log and indicates that the distribution is fat-tailed and well approximated by a scale-free function for at least two magnitudes of variation. This analysis indicates that long overlapping sequences are not rare. We discuss this result in section VI. The modal value of overlap length is 4 bp. We observe a similar overlap distribution when we restrict our analysis to conserved ORs as defined in Section 2 (blue line in bottom panel of Fig 2). This result decreases the likelihood that long overlapping sequences are an artifact of the annotation procedure.

Replicative benefits predict negative correlations among the frequency of overlapping sequences, the genome length and the noncoding DNA. The observation that *Thermotoga maritima *possesses both the genome with the smallest fraction of noncoding DNA and the largest fraction of genes involved in an overlap, suggests that a replicative compression argument might explain the variation in overlapping sequences in our database. In other words, a bacterium for which a short genome is important is expected to have a small fraction of noncoding DNA and a high fraction of overlapping genes. We are, in resorting to this hypothesis, for simplicity assuming that noncoding DNA is not carrying adaptive information and that a genome can experience a reduction in the noncoding portion without significantly influencing function.

**Figure 3:**
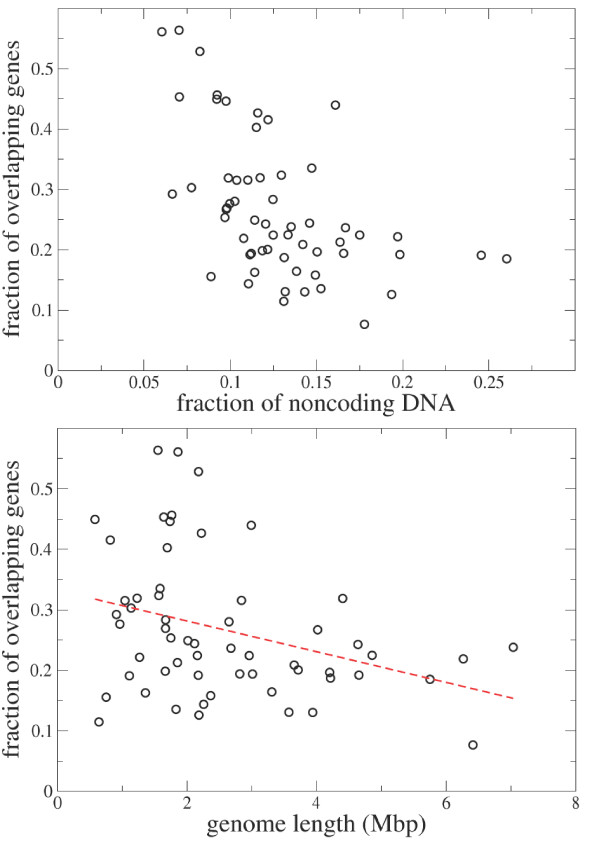
Top. Scatter plot of the fraction of genes involved in (at least) an overlap versus the fraction of noncoding genome. Bottom. Scatter plot of the fraction of genes involved in (at least) an overlap versus the genome length. The dashed line is a best linear fit. Both analyses are performed by considering only overlapping genes which are both annotated in the COG database.

We test this hypothesis by plotting in the top panel of Fig. 3 the fraction of genes involved in an overlap against the percentage of noncoding DNA. Each species is represented by a single strain and we do not correct for phylogeny. This is largely because not all species in the data set are well time-resolved and in addition we have no compelling model of genetic substitution within overlapping genes. Parsimony might be an acceptable first approximation, but without an exhaustive empirical study this remains conjectural. We do however, use parsimony to trace more coarse-grained features of overlapping genes, such as phase, in subsequent sections. The fraction of genes are here defined as the number of genes annotated as COG and involved in at least one overlap divided by the total number of genes annotated as COG. A statistically significant anticorrelation (*ρ *= 0.53, *p *= 10^-5^) is found indicating that when genome compression is important the overlap is high and the noncoding fraction is low (and vice-versa).

A smaller but statistically significant correlation (*ρ *= -0.33, *p *= 0.01) is observed between the fraction of genes involved in an overlap and total genome length (see bottom panel of Fig. 3). Here we reason that selective pressures for increased compression are favored in shorter genomes (in absolute terms), and hence the observed negative correlation between the genome length and overlap frequency. Of course, organisms cannot completely minimize their genomes as they need to retain the ability to synthesize essential proteins. This requirement establishes a lower bound on the correlation. Finally we observe a correlation coefficient of 0.32 between the fraction of noncoding genomes and the genome length (plot not shown).

**Figure 4:**
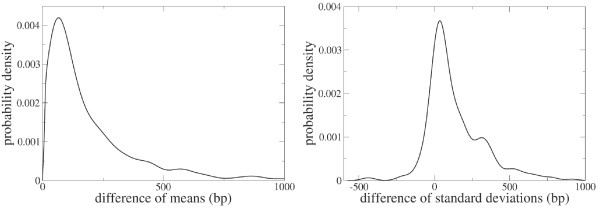
Left. Distribution of the differences between the mean sequence length of the overlapping genes and the mean sequence length of their orthologues that do not overlap. The set is comprised of the 290 COGs for which a *t *test rejects the null hypothesis that the mean length is equal in the two sets (overlapping and non overlapping). Right. Distribution of the differences between the standard deviations of the lengths of the overlapping genes and of the standard deviation of the lengths of the orthologous genes that do not overlap.

An additional way to investigate whether selection pressures for compression favor overlap is to compare the length of the overlapping genes to their orthologues which do not overlap. Under the hypothesis that a shorter genome is favored by selection one might expect that overlapping genes are shorter than their orthologues that do not overlap. For each of the 5412 COGs we extract all of the genes from the 58 genomes belonging to one COG and we divide this collection in two subsets, one containing those genes that contribute to an overlap and the other containing the orthologous genes that are not involved in any overlap. We then perform a *t *test with the null hypothesis that the mean length of the genes in the two subsets is equal. The requirement that there are at least two genes in both subsets decreases the number of COGs to 2059. For 290 (14.1%) COGs one must reject the null hypothesis that the two means are equal within 95% confidence. In all of these cases the mean gene length of the subset of overlapping genes is shorter than the mean gene length of the complement of orthologous, non-overlapping genes. The difference between mean gene length in the two subsets is quite large. The left panel of Fig. 4 shows the distribution of the differences between the mean sequence lengths in the two subsets. On average, overlapping genes are 220 bp shorter than their orthologues that do not overlap (the median value of the difference is 110 bp). The distribution of the differences between the standard deviations in lengths is fairly asymmetric. The right panel of Fig. 4 shows the distribution of the differences of the standard deviations in the two subsets. The distribution has more mass for positive values indicating that the sequence length of overlapping genes has a smaller dispersion than the sequence length of orthologous genes that do not overlap.

**Figure 5:**
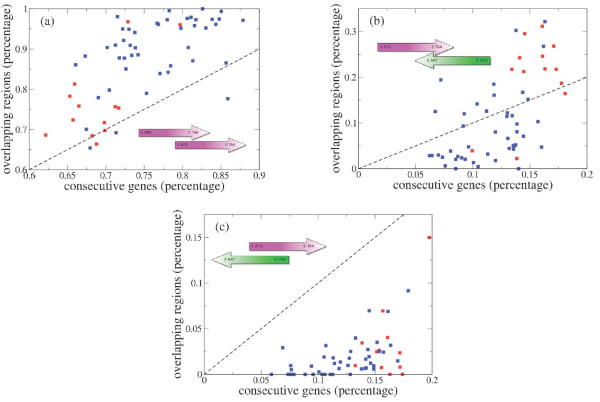
Percentage of overlapping genes in a given configuration (codirectional (a), convergent (b), divergent (c), see text) versus the percentage of consecutive genes in the same configurations to include non-overlapping genes. The dashed lines are the *y *= *x *lines and serves as a guide to the eye for testing the hypothesis that the two percentages are equal. The color code is red for Archaea and blue for Bacteria. The analysis is performed by considering only those overlapping genes annotated in the COG database. The arrows in each panel gives a visual representation of the different types of overlap. Magenta and green arrows indicate genes in the normal and complementary strand, respectively.

The hypothesis of immediate regulatory benefits requires that overlaps should preferentially be found on the same strand and be short. Given a pair of consecutive genes in a prokaryotic circular genome, they can occupy four possible orientations. When the two genes are in the normal strand or both are in the complementary strand (we call them codirectional), when the first is in the normal strand and the second in the complementary strand (convergent), and finally when the first is in the complementary strand and the second in the normal strand (divergent). If the two genes are overlapping, each of the 3 different configurations leads to a distinct type of overlapping sequences. The arrows inside the panels of Figure 5 give a graphical representation of the different overlap types.

We have investigated the joint probability of finding a pair of consecutive genes (not necessarily overlapping) in one of the three orientations described above. This property will prove to be important serving as a null hypothesis for comparison with the distribution of configurations of overlapping sequences. In a circular genome the number of convergent gene pairs needs to be exactly equal to the number of divergent gene pairs. In a linear genome one of the two can be at most larger than the other by one. Since the number of genes is quite large (of the order of thousands of genes) the percentage of convergent pairs is approximately equal to the percentage of divergent pairs.

For the full set of 58 species the percentage of codirectional sequences is larger than 50%. As has been observed in prior studies, this probably reflects the advantage of having genes in the same strand in order to coordinate the regulation of genes in operons [2]. Archaea have on average a smaller fraction of consecutive genes on the same strand when compared to Bacteria (see below the discussion of Figure 5). The differences between Archaea and Bacteria are probably related to the reduced incidence of operons in Archaea. In many prokaryotic species the percentage of codirectional sequences in the normal strand is close to the percentage of codirectional sequences in the complementary strand, indicating the absence of a functional preference for either one of the two strands [17].

We now compare the empirical frequencies of the different configurations of consecutive genes with the distribution of configurations for overlapping genes. Clearly there are 3 configurations of overlapping genes depending on the orientation of the two genes involved in the overlap. We will make use of the same terms, codirectional, convergent, and divergent, to indicate different configurations of overlapping genes. Figure 5 shows the percentage of the 3 configurations of overlapping genes in the 58 genomes versus the percentage of consecutive (non necessary overlapping) genes of the same type.

Assuming that the occurrence of an overlapping sequence was equally efficient in terms of the compression of information for the three possible configurations, one might expect that the probabilities for the 3 configurations of overlapping sequences were approximately equal to the probabilities for the 3 configurations of non-overlapping, paired genes. Figure 5 indicates that this is not the case. In fact, in the vast majority of cases, divergent overlapping genes are strongly underrepresented. Moreover, the convergent overlapping sequences are more common in Archaea (but also in the Chlamydiae and Cyanobacteriae) than in most Bacteria. The under representation of divergent overlapping sequences is probably the result of the necessity of encoding regulatory elements in the upstream region of genes for this configuration [17].

We test the null hypothesis that the configurations of overlapping sequences are distributed identically to the configurations of consecutive genes. In 53 cases the *χ*^2 ^test rejects the null hypothesis with 99% confidence. The 5 prokaryotes for which the null hypothesis cannot be rejected are *Halobacterium *(Archaea), *Mycoplasma penetrans*, *Ureaplasma urealyticum *(Firmicutes), *Buchnera aphidicola*, (Proteobacteria bacteria), *Treponema pallidum *(Spirochaetes). In summary the analysis of the frequencies of overlap configurations lends support to the immediate regulatory hypothesis.

**Figure 6:**
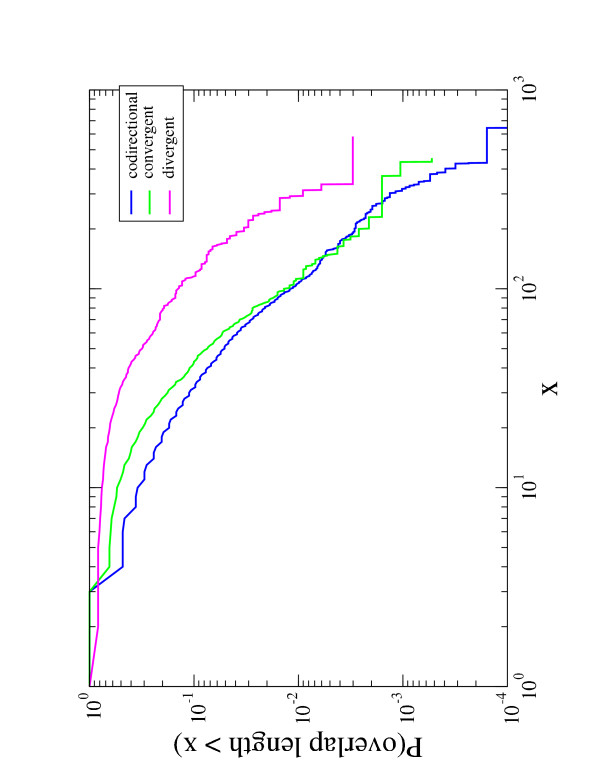
Cumulative density function of the length of the 3 configurations of overlapping sequences in our dataset. The color code is blue for codirectional (12, 737 sequences), green for convergent (1, 890 sequences), and magenta for divergent (331 sequences). The plot is double logarithmic and the analysis is performed by considering only overlapping genes which are both annotated in the COG database.

The second prediction of the immediate regulatory benefit hypothesis is that overlapping sequence should be short. The inset of the bottom panel of Figure 2 shows that, even if long sequences are frequently observed, the modal overlap length is 4 bp. This result can be better appreciated if one considers the overlap length distribution for the three different configurations discussed above. Figure 6 shows the cumulative density function of the length of the 3 types of overlapping genes. The figure indicates that codirectional and convergent overlapping sequences are similarly distributed, whereas the divergent sequences, that constitute a small fraction of the total overlapping sequences (see Fig. 5), are distributed in a different way.

A closer inspection of Fig. 6 illustrates the presence of discontinuities in the cumulative density functions at *x *= 4 for codirectional and convergent overlapping sequences. These jumps are a result of overlapping sequences of length 4 with a start (ATG) and a stop (TAA, TAG and TGA) codon. For the codirectional overlapping sequences, the tetramer ATGA contains the stop codon (TGA) of the first gene and the start codon (ATG) of the second gene. For convergent overlapping sequences, four tetramers CTAA, TTAA, CTAG, and TTAG are consistent with the termination of the two genes in opposite strands. Thus the abundance of short overlaps, and specifically those on the same strand and of length 4 bp, is an indication that regulatory genomic compression is a key factor in promoting short overlapping sequences.

Assuming that overlapping genes provide immediate regulatory benefits (for example when there is a short codirectional overlap) they can also create new challenges for the regulation of gene expression. Consider the case of convergent overlapping genes. One important mechanism of transcriptional termination in Prokaryotes is rho-independent termination. The signal for termination is a stable hairpin followed by a U-rich sequence. For *E. coli *approximately half of the transcriptional units are thought to be terminated by a rho-independent terminator [31]. For genes involved in a convergent overlap, the rho-independent terminators must be present in the coding sequence of one of the overlapping genes. With this constraint one might expect that the genes participating in convergent overlaps rarely make use of rho-independent motifs. We investigate this in the *E. coli *genome where 58 convergent overlaps are observed. We extract those genes that have a putative rho-independent terminator among those descibed in Ref. [31]. Somewhat surprisingly we find that in 19 overlaps one of the two genes makes use of a rho-independent terminator and in 2 overlaps, both genes have two (different) rho-independent terminators. In total only 23 (20%) of the 116 convergent overlapping genes have a rho-terminator. These results suggest that selection often succeeds in creating compressed sequences with dual coding and regulatory properties.

#### 3.1.1 Phylogenetic patterns in abundance statistics

**Figure 7:**
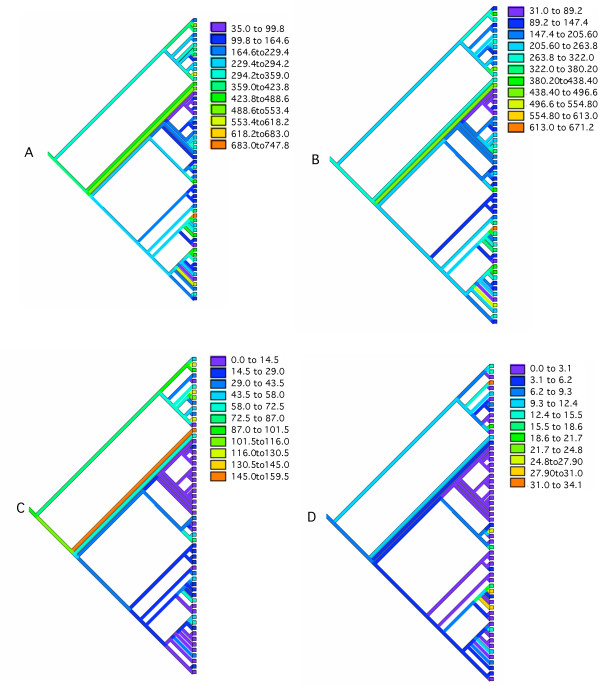
The overlapping phase relations traced through the prokaryotic phylogeny using squared-change parsimony and color-coded in each of the 4 trees. Trees are color-coded by the frequency of overlaps as indicated in the color-scale. A: Total number of overlapping genes, B: Total number of codirectional overlaps, C: Total number of convergent overlaps, D: Total number of divergent overlaps

Using squared-change parsimony ([32]) we have traced the total number of overlaps, in addition to the number of co-directional, convergent and divergent overlapping genes through the consensus Prokaryotic phylogeny illustrated in Figure 1. The results are shown in figure 7 where the color coding relates the numerical values as indicated in the accompanying color-scales. When it comes to the total number of overlaps there is no outstanding clade-level regularity other than the appearance of an elevated number of overlaps in the proteobacterial clades, the crenarchaeota and the euryarchaeota. Interestingly, based on the wide distribution of abundant overlaps over the tips of the tree, many of the more ancestral groups would seem to have possessed a higher than average number of overlapping genes than their descendants. Derived reduction in overlap is most pronounced in the firmicutes which possess a below average number of overlapping genes. Co-directional overlaps are fairly abundant across most species (other than the firmicutes), whereas convergent and divergent overlaps are restricted from most clades and are most common in the crenarchaeota and euryarchaeota. Exceptions to this pattern are found for the alpha-proteobacteria, where a few species, most notably the *Caulobacter sp. *shows a substantial, derived increase in the abundance of both convergent and divergent overlapping genes.

### 3.2 Phase Statistics

In order to test for variational benefits, above and beyond biases resulting from mutation, we investigate the statistical properties of different phases in which a given overlap can be found. For each configuration of overlapping sequence there are three possible phases, that is, ways of placing codon positions coupled to one another. Depending on which codon positions are complementary in an overlap, the effect of DNA mutations on the two genes can be different. We make use of the following notation for the different phases [17]. For codirectional overlaps we shall indicate the three phases as (123:123), (123:231), (123:312), depending on whether the first codon base of the first gene is also the first, second, or third codon base of the second gene. We use the same notation for codirectional sequences both in the normal and in the complementary strand, although the orientation of the two genes is opposite in the two cases. For overlapping genes of type convergent and divergent the three phases are (123:321), (123:132), and (123:213). As in the previous section we do not include embedded genes in order to avoid ambiguities. For each type of overlap, we compute the percentage of overlaps in each phase. Table 2 shows the result of this analysis. Before commenting on this table, recall that many overlaps have a length equal to 4, as a result of a match between start and stop codons or between two stop codons in complementary strands (see Fig. 6). In Table 3 we have repeated the analysis of the different phases by restricting the analysis to overlaps longer than 4 nucleotides. The comparison of Tables 2 and 3 illustrates the importance of excluding overlaps of length 4 bp from this analysis, as their high incidence biases the relative phase frequencies. Moreover, we find an inversion of phase occurrence in codirectional overlaps in Firmicutes as a consequence of the reduced occurrence of overlaps of length 4 in species belonging to this clade.

**Table 2:** Percentage of occurrence of different phases of overlap in Prokaryotes. Each line refers to a different type of overlap (codirectional, convergent, and divergent). The last column shows the total number of occurences for each overlap type.

	(123:231)	(123:312)	(123:123)	abs. number
codirectional	64	36	0	12737

	(123:132)	(123:321)	(123:213)	abs. number

convergent	32	21	47	1890
divergent	16	25	59	331

**Table 3:** Percentage of occurrence of different phases of overlap in prokaryotes conditioned to the fact that the overlap is longer or equal to 5 nucleotides. Each line refers to a different type of overlap (codirectional, convergent, and divergent). The last column shows the total number of occurencies for each overlap type.

	(123:231)	(123:312)	(123:123)	abs. number
codirectional	26	74	0	6130

	(123:132)	(123:321)	(123:213)	abs. number

convergent	49	33	18	1218
divergent	20	30	50	274

For codirectional overlapping genes longer than 4 bp the empirical analysis suggests that (i) there are essentially no overlaps of phase (123:123). This is reasonable as the stop codon of the first gene is translated in frame through the second gene and this would lead to a premature termination of transcription of the second gene. (ii) The phase (123:231) is less frequent than the (123:312) phase. This is true on average (Table 3) but it is also systematically true for all the genomes. This result is quite surprising as these two phases are in some sense indistinguishable from the statistical perspective of the distribution of paired nucleotides. In both phases a first nucleotide complements a third nucleotide, a first complements a second nucleotide, and the remaining pair comprises a second and a third nucleotide.

For overlapping genes on different strands (type convergent and divergent) we observe two different patterns. Convergent sequences are more frequently observed in the phase (123:132) whereas divergent sequences are more frequent in phase (123:213). It is worth noting at this point that the phase frequencies indicated in Table 2 and 3 are also observed when we restrict the analysis to those overlapping sequences shared by at least two species.

**Figure 8:**
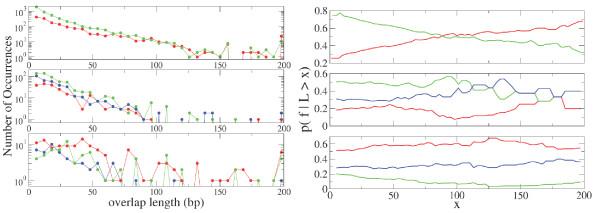
Left figure shows the histogram of the number of occurrences of the three phases in the overlap of a given type as a function of the overlap length. Right figure shows the probability of observing one of the three phases in the overlap of a given type conditioned on the restriction that the length of the overlap is longer than *x*. In each figure the three panels refer to codirectional (top), convergent (middle), and divergent (bottom) overlaps. The color code is (i) for codirectional overlaps (123:231) (red), (123:312) (green) and (123:123) (blue). (ii) for convergent and divergent overlaps (123:132) (green), (123:321) (blue) and (123:213) (red).

We now consider phase preference as a function of overlap length. We have analyzed the distribution of overlap lengths, investigating independently different phases of overlap. In the right panel of Figure 8 we show the histogram of the number of occurrences of different phases of overlap as a function of overlap length. Since fluctuations in the histogram are large, we investigate overlap length distribution for alternative phases using a different method. Specifically in the right panel of figure 8 we plot the quantity *p*(*f*|ℓ > *x*), the probability of observing one of the three phases *f*_1_, *f*_2_, and *f*_3 _in an overlap of a configuration (codirectional, convergent, and divergent) conditioned on the constraint that the overlap is longer than *x *base pairs. The three phases are plotted relative to their configuration through a normalization for each value of *x*, i.e. *p*(*f*_1_|ℓ > *x*) + *p*(*f*_2_|ℓ > *x*) + *p*(*f*_3_|ℓ > *x*) = 1. Interestingly, the curves for the codirectional overlapping sequences display a crossing at length ~75 base pairs. There are more than 300 codirectional sequences longer than 75 bp. For sequences shorter than this value the phase (123:312) is more frequent than the phase (123:231), whereas for longer sequences the order is reversed. Similarly for convergent sequences the conditional frequencies tend to equality for very large overlap. Finally for divergent overlaps, no crossing is observed.

#### 3.2.1 Phylogenetic patterns in phase statistics

**Figure 9:**
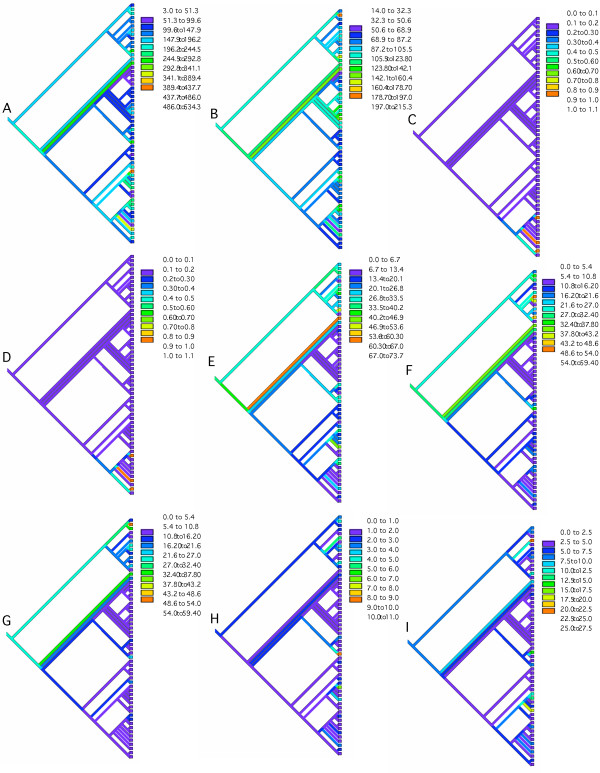
The overlapping phase relations traced through the Prokaryotic phylogeny and color-coded in each of the 9 trees are as follows: A: codirectional overlaps in phase (123:231), B: codirectional overlaps in phase (123:312), C: codirectional overlaps in phase (123:123), D: convergent overlaps in phase (123:213), E: convergent overlaps in phase (123:132), F: convergent overlaps in phase (123:321), G: divergent overlaps in phase (123:132), H: divergent overlaps in phase (123:213), I: divergent overlaps in phase (123:321).

In the preceding section we found that phase is strongly dependent on the polarity of transcription of the overlapping genes. We also observe a phylogenetic determinant (see Fig. 9). Among those species possessing co-directional overlap phase (123:231) the firmicutes and cyanobacteria are rare. Members of the Archaeota and Proteobacteria are better represented. For overlap phase (123:312) there are many firmicutes. There are very few species of any clade possessing phase (123:123). For convergent overlap the Archeaota are the most abundantly represented clade for both (123:132) and (123:321) phases. The two species, *Aquifex aeolicus *and *Thermotoga maritima *also possess a high frequency of genes in these two phases. The species *Pseudomonas aeruginosa *has a very high abundance of phase (123:213). Among the remaining clades only a few cyanobacteria show evidence of a convergent overlap above the prokaryotic average. For divergent overlaps the pattern is very similar to the convergent case with only a few species behaving contrary to their clade, in particular *Mycobacterium tuberculosis *has an elevated frequency of (123:213) in comparison to the Actinobacteria, whereas *Caulobacter crescentus *is elevated in (123:321) in comparison to the remaining Alpha-proteobacteria. Overall, co-directional overlap would seem to be a more ancestral form of organization than convergent and divergent where we find a larger number of unique derived instances in the species distribution.

## 4 The 3-fold evolutionary dynamic

Microbes are distinguished by their enormous abundance and by the rapid rate at which they replicate. Both of these features make demands on the organization and regulation of the genome. Competition in large population sizes typically selects for fast and efficient replication and this in turn requires that protein synthesis is rapid and low cost. One very general mechanism for propagating information at a high rate is data compression, a technique indispensable in computer technology, whereby redundancies in a body of information are minimized and where common messages are given short description lengths [6]. In this study we have analysed an evolved form of data compression, namely, the properties of overlapping genes in a large set of 58 fully sequenced prokaryotic genomes. Both prokaryotes and phage are known to exploit overlap extensively [11]. We have organized our research into three sets of evolutionary questions. First: how do overlaps come about and by what mutational process, and how do these mutations bias the conserved overlap lengths and phases that we observe in the database? Second: for highly conserved patterns of overlap shared by multiple lineages, how can we explain regular patterns in the data accruing through immediate benefits to an individual genome? Third: among those patterns of overlap that do not relate to immediate benefits (rates of replication or expression) but do have implications for population variability, is the data consistent with neutrality, redundancy or amplification?

### 4.1 Stochastic Origination

We corroborate earlier studies showing that the modal number of overlapping nucleotides is equal to 4 and that these 4 nucleotides frequently overlap initiation and termination codons of contiguous genes, or the termination codons of complementary overlaps. This suggests that neutral point mutation is an important mechanism for promoting overlapping genes. We directly test the stop codon mutation (SCM) mechanism by performing a detailed case study of *Escherichia coli *overlapping sequences.

The SCM mechanism promotes the formation of an overlap through a single nucleotide mutation within a stop codon. Consider two consecutive, non-overlapping genes in the same or in complementary strands. The stop codon of one of the two genes mutates (or is deleted) and this leads to a read-through until a new stop codon is encountered in the down-stream gene creating a sequence of overlap. Let us assume as a null hypothesis that the function of the new gene is not altered by the elongation of the first sequence. Can we estimate the expected length and phase properties of the overlap? The probability that the overlap length is longer than *x *codons, i.e. *y *= 3*x *nucleotides, is equal to the probability that all *x *codons encountered in the read-through are different from a stop codon. We indicate with *p *the probability that a codon is different from a stop codon, and we conclude that *p*(*overlap length *> 3*x*) = *p*^*x *^i.e.

p(overlap length>y)=py/3=ey3ln⁡p≡e−y/yc
 MathType@MTEF@5@5@+=feaafiart1ev1aaatCvAUfKttLearuWrP9MDH5MBPbIqV92AaeXatLxBI9gBaebbnrfifHhDYfgasaacH8akY=wiFfYdH8Gipec8Eeeu0xXdbba9frFj0=OqFfea0dXdd9vqai=hGuQ8kuc9pgc9s8qqaq=dirpe0xb9q8qiLsFr0=vr0=vr0dc8meaabaqaciaacaGaaeqabaqabeGadaaakeaacqWGWbaCcqGGOaakcqWGVbWBcqWG2bGDcqWGLbqzcqWGYbGCcqWGSbaBcqWGHbqycqWGWbaCcqqGGaaicqWGSbaBcqWGLbqzcqWGUbGBcqWGNbWzcqWG0baDcqWGObaAcqGH+aGpcqWG5bqEcqGGPaqkcqGH9aqpcqWGWbaCdaahaaWcbeqaaiabdMha5jabc+caViabiodaZaaakiabg2da9iabdwgaLnaaCaaaleqabaWaaSaaaeaacqWG5bqEaeaacqaIZaWmaaGagiiBaWMaeiOBa4MaemiCaahaaOGaeyyyIORaemyzau2aaWbaaSqabeaacqGHsislcqWG5bqEcqGGVaWlcqWG5bqEdaWgaaadbaGaem4yamgabeaaaaaaaa@5DC3@

where *y*_*c *_≡ -3/ln(*p*). This argument illustrates that one might expect from this simple model an exponential distribution of overlap lengths. This is not observed in our data. In Figure 2 we illustrate the empirical overlap length distribution and the best fit of the distribution to an exponential function exp(-*ax*) with the same mean. The discrepancy between the two curves indicates that either the SCM is not the only mutational mechanism generating sequences or that some additional selective processes have filtered the variation.

The SCM mechanism also makes predictions about the expected distribution of overlap phases generated by a stop mutation. As a result of the bias in the codon distribution of a species, it is more likely that the read-through process finds a new stop codon in some phases over others. Consider two codirectional genes in an *E. coli *genome and further suppose that the stop codon of an upstream gene mutates with the read-through continuing until a new stop codon is encountered in the downstream gene. In *E. coli *there are three stop codons, TAA, TAG and TGA. We indicate with *P*(*XYZ*) the empirical frequency of a codon *XYZ*. By assuming that consecutive codons behave independently, the probability of finding a stop codon in a given phase can be calculated. For example, the probability of finding *TGA *in phase (123:312) is calculated via the sum:

∑X∈A,C,G,TP(XTG)∑Y,Z∈A,C,G,TP(AYZ).
 MathType@MTEF@5@5@+=feaafiart1ev1aaatCvAUfKttLearuWrP9MDH5MBPbIqV92AaeXatLxBI9gBaebbnrfifHhDYfgasaacH8akY=wiFfYdH8Gipec8Eeeu0xXdbba9frFj0=OqFfea0dXdd9vqai=hGuQ8kuc9pgc9s8qqaq=dirpe0xb9q8qiLsFr0=vr0=vr0dc8meaabaqaciaacaGaaeqabaqabeGadaaakeaadaaeqbqaaiabdcfaqjabcIcaOiabdIfayjabdsfaujabdEeahjabcMcaPmaaqafabaGaemiuaaLaeiikaGIaemyqaeKaemywaKLaemOwaOLaeiykaKIaeiOla4caleaacqWGzbqwcqGGSaalcqWGAbGwcqGHiiIZcqWGbbqqcqGGSaalcqWGdbWqcqGGSaalcqWGhbWrcqGGSaalcqWGubavaeqaniabggHiLdaaleaacqWGybawcqGHiiIZcqWGbbqqcqGGSaalcqWGdbWqcqGGSaalcqWGhbWrcqGGSaalcqWGubavaeqaniabggHiLdaaaa@542D@

The predicted probabilities are shown in Table 4.

**Table 4:** Percentage of occurrence of different phases of overlap in *E. coli*. The table includes the percentage of observed overlap longer than 4 bp in different orientations and phases and compares these empirical values with the percentages expected by the stop codon mutation hypothesis (SCM).

	codirectional			convergent		
	(123:231)	(123:312)	(123:123)	(123:132)	(123:321)	(123:213)
observed	32	68	0	46	32	22
SCM	54	46	0	39	34	27

Table 4 shows the frequencies of the observed phases of overlapping sequences for *E. coli *and the expected frequencies according to the SCM mechanisms. For codirectional overlapping genes we find that sequences longer than 5 bp in phase (123:231) comprise 32% and phase (123:312) 68%. The SCM mechanism predicts approximately equal frequencies for the two phases (54% and 46%, respectively). This discrepancy suggests that the stop mutation mechanism cannot be the only mechanism promoting overlapping sequences in the same strand. In Figure 8 we observe that short sequences on the same strand are more frequent in (123:312) phase, whereas long sequences are more frequent in (123:231) phase. Table 4 suggests that long sequences on the same strand are more likely to be generated by the SCM mechanism.

The origin of convergent sequences can be tested only with the SCM mechanism for which a simple statistical model can be derived. Table 4 shows a good agreement between the observed and the expected frequencies, suggesting that SCM is the most common mechanism generating this type of overlapping sequence. This result is in agreement with [33] where the convergent sequences of *Mycoplasma *appear to be generated by a stop mutation mechanism.

A related question is whether the usage of stop codons is the same for overlapping and non-overlapping genes. To this end we have divided the set of genes into three subsets: (i) Genes with a 3' end not involved inn the overlap (but where the 5' end can be overlapping). (ii) Genes with a 3' end involved in a codirectional overlap (where the gene is upstream of the overlap). (iii) Genes with a 3' end involved in a convergent overlap. We then counted the total number of the three different stop codons for the three subsets. The left part of Table 5 shows the fraction of genes in the three subsets for *E. coli*. It is evident that the usage is quite different in the different sets. Part of this difference could be a direct result of short overlaps. For example in set (ii) (codirectional overlaps), TGA could favored through a matching of stop and start codon at the ATGA tetramer. Table 5 set (ii) has a percentage of TGA of 68% compared to the 24% for non overlapping genes. To take into account this bias we now only consider overlaps longer than 4 bp where a matching of start and stop codons is excluded. The right part of Table 5 shows the frequencies in this restricted set. The stop codon usage is now more similar for across the sets.

**Table 5:** Percentage of stop codons in the three subsets for *E. coli*. For the definition of subsets, see the text. The left half refers to all possible overlap lengths, whereas the the right part of the table only includes overlaps longer than 4 bp.

	*TAA*	*TAG*	*TGA*	*TAA*	*TAG*	*TGA*
set (i)	68	8	24	68	8	24
set (ii)	28	4	68	54	8	38
set (iii)	63	19	18	49	19	32

In order to determine the statistical significance of the observed differences in stop codon usage for each genome we perform *χ*^2 ^tests. Specifically we took take (i) as a reference and tested whether set (ii) and set (iii) have the same distribution of stop codon usage as set (i). We independently analyze the set including all possible overlap lengths from the set of overlaps longer than 4 bp. From the 58 genomes we are able to perform the test on at most 52 because the remaining 6 bacteria use only 2 different stop codons. We adopt a 99% confidence for significance. When we consider the set of all possible overlap lengths we find that for 98% of the genomes (51/52) we are reject the hypothesis that set (ii) has an equal stop codon distribution as set (i) and for 48% (19/40) genomes we reject the hypothesis that set (iii) has an equal stop codon usage as set (i). For the set of overlaps longer than 4 bp we find that for 65% (34/52) of the genomes we reject the hypothesis that set (ii) has an equal stop codon distribution to set (i) and for 35% (13/37) genomes we reject the hypothesis that set (iii) has an equal stop codon usage to set (i).

In conclusion, when taking into account genome individuality and possible biases introduced by a coupling between start and stop (or two stop) codons, we find that stop codon usage is different for overlapping and non overlapping genes. This effect is stronger for codirectional than for convergent genes. This difference could provide some useful insights into the the origin of overlapping genes.

### 4.2 Immediate Benefits

While stochastic processes involving mutation and frame-shifts can explain some of the variation in the data, they are not able to explain certain widespread, conserved features. We observe that short overlapping genes frequently arise on the same strand, presumably to encourage translational coupling among coregulated genes in an operon. We also find long overlaps in complementary strands, which would serve to reduce the number of nucleotides required to encode two or more proteins.

In prokaryotes genomes, around 30% of all genes are involved in some degree of overlap, reaching an extreme value of 56% in the species *Aquifex aeolicus *and *Thermotoga maritima*. The mean length of the overlapping sequences is 26 base pairs, whereas the modal value is 4 base pairs. These 4 base pairs can occur as overlaps on a single strand in which case the termination and initiation codons overlap, or in complementary strands in which case complementary termination codons overlap. We find that a greater proportion of overlaps are in a single strand. These observations argue in favor of overlap promoting regulatory compression within co-regulated gene complexes of a strand, in particular within operons. This is the position advocated in recent research [10] emphasizing the high frequency of 4 nucleotide overlaps. Regulatory compression is achieved by having the overlapping sequence play a dual termination and initiation role, and eliminates the time delay associated with the initiation phase of protein synthesis by promoting translational coupling [34,20]. Futhermore, the importance of regulation in overlap is suggested by the observation that the least frequent of the overlapping configurations is divergent, in which the 5' upstream portion of each gene is overlaping and the 3' downstream portion non-overlapping. This configuration potentially places both the Shine-Delgarno motif and the initiation codon in the overlapping portion of the sequence. Since both of these require specific motifs, they constrain the evolution of their complementary genes in the overlap. In the non-overlapping paired gene control, convergent and divergent are indistinguishable. Thus regulatory elements constrain the prefered overlapping configurations. An additional observation is that convergent and divergent overlaps are both more frequent in the Archaea than in the Bacteria, whereas in the non-overlapping controls these domains (or superkingdoms) are indistinguishable. Recent results suggest an additional form of regulation, whereby overlapping genes in complementary strands are capable of reciprocal regulation through formation of complementary anti-sense transcripts in prokaryotes [5] and also somewhat surprisingly in eukaryotes [7] and vertebrates [21].

While these data corroborate an important function and constraint on overlap derived from regulatory considerations, we also find evidence for replicative compression. Long overlapping sequences occur frequently (the mean overlap length is significantly larger than the mode), and very often appear on complementary strands which are independently regulated. The importance of replicatory compression is further supported by the fact that the shorter genomes also have a higher proportion of overlapping genes and a reduced fraction of non coding DNA. Shorter genomes also possess a greater proportion of overlapping genes of shorter length than their non-overlapping orthologues (Figure 4). The tendency of organelle and microbial genomes to eliminate all extraneous nucleotide material has been termed genomic reduction, and is well documented in mitochondria and virus genomes where pressures on replication rates are at a premium [35]. A similar tendency has been recently reported for a free-living prokaryote [36]. These results together with our findings are suggestive of a role for overlap promoting genomic reduction.

### 4.3 Variational Benefits

Overlapping genes also vary in their preferred phases; when the overlapping genes are independently translated, mutations of large effect are encouraged. Contrariwise, longer overlapping sequences tend to favor phases where redundancy is maximized.

To address the questions of the variational properties of overlapping sequences, we need to examine the phase of overlapping genes. As a result of the triplet assignment rules of the canonical genetic code, genes can overlap in 6 different phases. In genes that are encoded in a single strand, these can be written as (123:123), (123:312) and (123:231) [9]. These describe the virtual base pairings in the context of the overlapping codons. In the first phase, the bases are perfectly aligned, and this phase is almost never observed. In the second phase, the most significant base (MSB) of the upstream gene (base 2) pairs with the intermediate significant base (ISB) of the downstream gene (base 1), while the least significant base (LSB = base 3), pairs with the MSB. We use MSB, ISB and LSB to describe the relative degeneracy of codon positions. Notice that mutations to the genes are going to be asymmetrical with respect to strand, with synonymous mutations to the LSB of the upstream gene causing a change at the MSB of the downstream one, whereas mutations to the LSB of the downstream gene alter the ISB of the upstream. In the third phase, the situation is again asymmetrical, but in this case the properties of the upstream gene are those of the downstream and vice versa.

We find that for codirectional overlaps, (123:312) is the most common at shorter overlaps, whereas (123:231) becomes more frequent at the longest overlaps. Can we understand this in terms of mutational susceptibility? For the sake of argument and without loss of generality, we make the following assumptions. The probability per nucleotide per generation of mutation is *μ*. The probability that the mutation is non-synonymous at the MSB is equal to 1. The probability of non-synonymous mutation at the ISB is equal to *ε*, where 0 <*ε *< 1, And the probability at the LSB is equal to 0. Let us further assume that the effects of mutations on phenotypes are multiplicative, such that the phenotype of a genome with *i *mutations is given by (1 - *p*)^*i*^, where *p *is the average phenotypic effect of an amino acid substitution. This provides us with a metric with which we can compare phenotypes to the wildtype which assumes a phenotypic value of 1. If we assume a single point mutation to a sequence, then the average phenotype of a genome with an overlapping proportion of nucleotides *ρ *in phase (123:312), can be calculated as follows. Mutations at the first nucleotide position of an overlapping triplet lead to substitution probabilities in the two genes, *ε *and 0, at the second position 1 and *ε*, and the third position 0 and 1. The expected phenotype of a sequence with a single mutation falling in the overlapping sequence with a per genome per generation probability *ρμ*, is then equal to:

*F*_+1 _= 13
 MathType@MTEF@5@5@+=feaafiart1ev1aaatCvAUfKttLearuWrP9MDH5MBPbIqV92AaeXatLxBI9gBaebbnrfifHhDYfgasaacH8akY=wiFfYdH8Gipec8Eeeu0xXdbba9frFj0=OqFfea0dXdd9vqai=hGuQ8kuc9pgc9s8qqaq=dirpe0xb9q8qiLsFr0=vr0=vr0dc8meaabaqaciaacaGaaeqabaqabeGadaaakeaadaWcaaqaaiabigdaXaqaaiabiodaZaaaaaa@2EA0@[(1 - *p*)*ε *+ (1 - *ε*) + (*ε*(1 - *p*)^2 ^+ (1 - *ε*)(1 - *p*)) + (1 - *p*)]

A similar value can be calculated for (123:231):

*F*_+2 _= 13
 MathType@MTEF@5@5@+=feaafiart1ev1aaatCvAUfKttLearuWrP9MDH5MBPbIqV92AaeXatLxBI9gBaebbnrfifHhDYfgasaacH8akY=wiFfYdH8Gipec8Eeeu0xXdbba9frFj0=OqFfea0dXdd9vqai=hGuQ8kuc9pgc9s8qqaq=dirpe0xb9q8qiLsFr0=vr0=vr0dc8meaabaqaciaacaGaaeqabaqabeGadaaakeaadaWcaaqaaiabigdaXaqaaiabiodaZaaaaaa@2EA0@[(*ε*(1 - *p*)^2 ^+ (1 - *ε*)(1 - *p*)) + (1 - *p*) + (1 - *p*)*ε *+ (1 - *ε*)]

Where *F*_+*i *_indexes a positive translational offset *i *in the same strand. These are identical and hence on the basis of the changing phenotypes induced by mutations to overlapping sequences, there is no reason to expect any difference in the frequencies of these two phases.

What then is the cause of the observed difference in phase frequency? We discussed in Section VI a possible explanation in terms of mutational mechanisms producing overlapping sequences. We also find that for those overlaps longer than 150 bp, the frequencies of the codirectional phases invert. We have yet to understand this trend (see also Section VI).

In the convergent and divergent configurations two regimes are observed. For overlaps shorter than ~50 bp the most common phase is the (123:132) (see Fig. 8). For longer overlaps, the phase (123:213) becomes increasingly more common (together with (123:321)).

We can write down the expected phenotype functions for each of the three phases negatively offset in the complementary strand:*F*_-2_, *F*_-1_, *F*_-0_, corresponding to the phases (123:132), (123:213), (123:321):

*F*_-2 _= 13
 MathType@MTEF@5@5@+=feaafiart1ev1aaatCvAUfKttLearuWrP9MDH5MBPbIqV92AaeXatLxBI9gBaebbnrfifHhDYfgasaacH8akY=wiFfYdH8Gipec8Eeeu0xXdbba9frFj0=OqFfea0dXdd9vqai=hGuQ8kuc9pgc9s8qqaq=dirpe0xb9q8qiLsFr0=vr0=vr0dc8meaabaqaciaacaGaaeqabaqabeGadaaakeaadaWcaaqaaiabigdaXaqaaiabiodaZaaaaaa@2EA0@[(1 - *ε*)^2 ^+ 2(1 - *ε*)*ε*(1 - *p*) + *ε*^2^(1 - *p*)^2 ^+ 2(1 - *p*)]

*F*_-1 _= 23
 MathType@MTEF@5@5@+=feaafiart1ev1aaatCvAUfKttLearuWrP9MDH5MBPbIqV92AaeXatLxBI9gBaebbnrfifHhDYfgasaacH8akY=wiFfYdH8Gipec8Eeeu0xXdbba9frFj0=OqFfea0dXdd9vqai=hGuQ8kuc9pgc9s8qqaq=dirpe0xb9q8qiLsFr0=vr0=vr0dc8meaabaqaciaacaGaaeqabaqabeGadaaakeaadaWcaaqaaiabikdaYaqaaiabiodaZaaaaaa@2EA2@[(1 - *ε*)(1 - *p*) + *ε*(1 - *p*)^2^]

*F*_-0 _= 13
 MathType@MTEF@5@5@+=feaafiart1ev1aaatCvAUfKttLearuWrP9MDH5MBPbIqV92AaeXatLxBI9gBaebbnrfifHhDYfgasaacH8akY=wiFfYdH8Gipec8Eeeu0xXdbba9frFj0=OqFfea0dXdd9vqai=hGuQ8kuc9pgc9s8qqaq=dirpe0xb9q8qiLsFr0=vr0=vr0dc8meaabaqaciaacaGaaeqabaqabeGadaaakeaadaWcaaqaaiabigdaXaqaaiabiodaZaaaaaa@2EA0@[2(*ε*(1 - *p*) + (1 - *ε*)) + (1 - *p*)^2^]

After some algebra we find that,

*F*_-1 _≤ *F*_-2 _≤ *F*_-0_

This states that the phenotypic effect associated with phase (123:213) is the least robust. This is expected because this phase couples the MSB of one gene with the ISB of the other. Thus two out of three three times a mutation is non synonymous in at least one gene and the mutation is non synonymous in both genes with probability 2*ε*/3. This result helps us to understand why phase (123:213) is the least frequently observed (Table 3). The frequencies of the other two phases are in contrast with what expected from Eq. 8. In fact Eq. 8 suggests that phase (123:321) (corresponding to *F*_-0_) should be more frequent than phase (123:132) (corresponding to *F*_-2_) whereas Table 3 shows that the the reverse is observed in real genomes.

## 5 Conclusion

The analysis of overlapping sequences indicates that more than one mechanism is likely to be responsible for the origin and maintenance of these genomic traits. Not only do overlapping genes preserve features relating to their mutational origin, but secondary features relating to ongoing selection pressures. When two genes are on the same strand a mutation in the stop codon seems to be largely responsible for long overlaps. When two genes are in different strands the stop codon mechanism helps in explaining the abundance of different phases but we require additional selective hypotheses to explain phase variation. Our essential conclusions can be stated as follows:

1. When correcting for codon bias, both short overlaps – modal length 4 – and single strand usage can partly be accounted for in terms of the stochastic process of mutations to stop and start codons. This suggests a role for neutral processes in promoting gene overlap.

2. The preponderance of short overlaps in operons suggests an important role for translational coupling promoting overlapping sequences for efficient gene regulation. This is evidence for selection promoting regulatory compression through overlap.

3. Selection for genome minimization can be gleaned from shorter genomes containing a larger frequency of overlapping genes whose average length is less than that of their non-overlapping orthologues. This is evidence for selection promoting replicative compression through overlap.

4. The most common overlap phases promote redundant base pairings that can not be explained in terms of neutral mutations in the presence of codon bias. This suggests a role for selective contraints on coding sequences, promoting greater mutational robustness.

5. The persistence of many non-redundant phases in long sequences suggests some selection pressures for amplifying the effects of point mutations in these genes. This could reflect either selection for highly efficient purging of deleterious mutations or a mechanism for promoting high diversity under variable conditions.

6. Overlap length is a strong predictor of some overlap phases but this variation can not be explained in terms of the redundancy properties of each phase – both long and short phases can have the same error-buffering properties.

7. Long overlaps are a feature of many prokaryotic species and manifest a tendency towards a reduction in abundance over the course of evolution. This suggests the hypothesis that overlapping genes were a more common feature of ancestral prokaryotes.

8. There is considerable clade variation in phase preference, but very few species ever make use of the more mutationally deleterious phases.

9. Co-directional overlapping genes appear to be an ancestral genome trait whereas convergent and divergent configurations appear to be more derived.

These data collectively illustrate how much information can be compressed into a genome by exploiting the codon-based triplet code, and how variation in compression phases exists in relation to different selection pressures and modes of gene origination. Overlap is just one of several mechanisms organisms employ to create one to many maps from transcription to translation overcoming the collinearity constraint (1-gene 1-protein). Some alternatives are RNA editing [37] and post translational procesing. An additional source of multiple-coding, at least for recognition sites, derives from pressures reducing mutation load at high recombination rates [38]. In all cases, the combinatoric flexibility afforded by the transcription and translational mechanisms allow for adaptive information to be added to that provided by the underlying linear sequence. The linear sequence is revealed to be an expedient structure for storage purposes rather than a true measure of the total information content of the genome.

## Reviewers' comments

### Reviewer's report 1

Eugene Koonin, National Center for Biotechnology Information National Library of Medicine National Institutes of Health.

1. Overlapping genes are an old and appealing topic, tracing back to the startling discovery of 'genes within genes' in small bacteriophages by Sanger et al. back in 1977. However, the subsequent history of the study of this phenomenon has been somewhat anticlimactic because it turns out that long overlaps are, after all, not that common in cellular life forms (or even in viruses with larger genomes), and the longest ones reported have the unpleasant habit to go away as artifacts. However, the apparent lack of a truly essential biological role of overlaps – beyond very short overlaps involved in regulatory compression as discussed in the present paper – is not to diminish their theoretical interest. Even if the sequences of overlap are rather short, they do carry two messages in the same string of nucleotides, and an extensive analysis of such sequences on genome scale has the potential to reveal aspects of selection and neutrality that escape our attention in the study of "normal" genes.

The paper of Lillo and Krakauer is, to my knowledge, the most comprehensive and nuanced analysis of this kind to date, and beyond any doubt, will be a useful addition to the literature. Of course, I do have a variety of comments that might be of some use for revision or could just help the interested reader to become better oriented in this rather complex tangle of problems.

2. First a couple of very general issues. The work would greatly benefit from a more extensive and more explicit analysis of the evolutionary conservation of overlapping regions (ORs). This would make a lot of sense both methodologically, by increasing the reliability of the results, and substantively. Indeed, it is interesting to see how the evolutionary conservation varies among different orientations and phases of ORs, are there some ORs that are conserved in a broad range of prokaryotes, and more questions like that.

More or less along similar lines, it would make sense to present more information on possible lineage-specific trends in ORs. Is there some interesting biology here or are the characteristics of ORs just a function of genome size and gene density? If it is the latter, it is worth illustrating and stating explicitly. All the more so if it is the former.

*As we show in this paper, using some careful controls, long overlaps are in fact far more common than is thought. While short overlaps can be reasonably easily explained in terms of an ongoing legacy of neutral mutations, modest genome minimization and regulatory compression, long overlaps make a strong case for replicative compression. As is shown in figure *2*, the length distribution function indicates clearly how the longest overlap lengths exceed the expectations of the an exponential distribution with the same mean.*

With regard to lineage-specific trends, we include an explicit phylogenetic component by tracing several features of overlapping genes through a consensus prokaryotic phylogeny making use of squared change parsimony. This allows us to track varying degrees of conservation, and to identify lineages in which changes to the genes have been more recent or derived. We also consider a more restricted dataset including only overlaps formed by pairs of genes that are conserved in at least two species and in this way seek to minimize curatorial artifacts.

3. In the Background section, the authors discuss the mutational origins of overlaps and the interplay of neutral or adaptive processes in their evolution. However, I think it is rather important to explain right away the range of the phenomenon and to make distinction between viral overlapping genes where many occasions of actual "genes within genes" and prokaryotic overlaps that are (predominantly) very short.

We elected to not treat cases of embedded overlapping genes in this study. This is because we sought those cases where controls based on non-overlapping orthologues could be used in the analysis. Embedded genes are a fascinating topic as they often make additional demands on the post-transcriptional machinery, and as stated, are used extensively in viruses.

4. Variational benefits...this is an old, somewhat tired issue on the reality of "evolution of evolvability", evolution having no foresight etc etc. The authors present this as a fully legitimate, regular evolutionary force. Perhaps, some extra caution and more discussion are due.

We have pointed out that variation in phase can not be explained exclusively in terms of either regulatory or immediate benefits. There is an ongoing effect of mutational origin on the statistics of phase usage, but this is also unable to explain a significant portion of the variation. What does seem more likely is that phases that are preferred have mutational properties that are either conservative – more redundant, or are amplifying – increase the amino acid replacement probability. Both of these strategies are consistent with robustness arguments, in which genomes are either buffered from genetic variation or more efficiently purged in a clonal quasispecies. It is also possible of course that increased variation can come under positive selection, in which case this would constitute an evolveability hypothesis. With the current evidence we are unable to discriminate between the previous possibilities and we do not use the term evolveable although the hypothesis remains very much in play. We think that evolveability could be particularly interesting in microbes where population sizes are typically large and where indirect selective effects are thus able to exert a significant influence on adaptation. The existence of the mutator genotypes provide compelling evidence for this possibility.

5. The issue of selection for genome compression – not regulatory compression (with which I have no problem) but replication rate. This is very obvious and one of the first things that comes to mind when one considers the raison d'etre for overlaps. But is it real or, at least, is it particularly important and general? How much sequence can be actually saved through overlaps? Fig. 2a shows values > 10 kb for 5 genomes; whether this is a lot or not really depends on the size of the respective genomes (by the way, is it worth to show the same data after normalizing by genome size?) One can sort of get the hang of it by comparing Fig. 2a and Fig. 2b but it is not, exactly, straightforward. In any case, for the great majority of the genomes, the total length of the overlaps is much less. I understand it is no easy question but could there be any way to assess the selective advantages conferred by this amount of compression against the obvious disadvantages of overlaps (assuming they are not subject to other types of selection)? Also, if compression is so important, why no genes within genes? We know from viruses that this is not impossible.

This is now dealt with fairly explicitly in the text and the conclusions. The question of the magnitude of replicative benefit, ideally measured in terms of replication rates in culture, is difficult to quantify using only published sequence data. Having said this, it is instructive to note that around 3% of all genes are involved in overlap with a mean overlap length of 26 base pairs. In some species this percentage can be over 50%. The sum of all overlapping regions is on average 0.2% of the total genome length.

6. The rather notorious issue of long overlaps. It is possible that I am overly cautious but I am worried over the right tail of the distribution in Fig. 1b. Clearly, there are only a few points in this area, and even a small number of artifacts would sway the curve away from the exponent. At least, I think this issue should be given more attention.

*In the revised version we give more attention to this issue by considering a set of highly conserved ORs. See point 4 above and the new panel in Fig. *2.

7. The explanation of the striking under-representation of divergent overlaps given on p. 11 (and in ref. [25]) is, probably, correct. The point, I believe is that the constellation of regulatory elements that are required for the initiation of both transcription and translation is much more demanding than that required for termination. Hence the strong purifying selection against divergent but not so much against convergent overlaps. By the way, an interesting thing to check: are convergent overlaps in prokaryotes seen primarily in genes with rho-dependent or rho-independent termination? Sequence requirements for the two are very different.

We discuss this interesting problem in relation to the rho-independent terminators and convergent overlaps in E. coli. As the text indicates at least for E. coli there remains a relatively large number of sequence-dependent termination motifs even in overlapping sequences.

8. In the discussion of phase frequencies – relating to the issue of long overlaps once again. I am very worried about the "crossing" at length 75. How many points there, after all?

In the revised version we make this number clear and include the sentence 'There are more than 300 codirectional ORs longer than 75 bp'

9. In the conclusions it would be desirable to indicate that, alas, there is no good way to distinguish between the adaptationist and neutral explanations or any combination thereof. Under these circumstances, is it not prudent to take the neutral explanation as the null hypothesis?

As the paper shows there seems to be evidence for all three kinds of evolutionary explanation. While we agree that neutral hypotheses constitute an appropriate null model, we do find many patterns at odds with neutrality, from the most modest 4-base pair overlaps in operons, through to variation in phase preference in long overlapping sequences. We have tried to make these distinctions as clear as possible.

### Reviewer's report 2

Martijn A. Huynen, Ph.D. Center for Molecular and Biomolecular Informatics Nijmegen Centre for Molecular Life Sciences Radboud University Nijmegen Medical Centre

1. taxonomy: The position of the hyperthermophilic bacteria is, in my opinion, not resolved. Gene content (Dutilh, JME 2004) and indel analyses (Gupta) put Aquifex with the Proteobacteria, or at least at their root. Proteobacteria, and Thermotoga with the Firmicutes. That position of Aquifex would fit "better" their high level of overlapping genes which they share with the Proteobacteria. In line with this, I would be careful with the remark about the primitiveness of the overlapping organization, as you are, as far as I understand also referring to your results on the Archaea (not Archaeota) here. And there is no reason to assume that they are primitive.

We have endeavored to be cautious in the interpretation of the phylogenetically reconstructed patterns, largely because the status of the phylogeny remains somewhat ambiguous. Our tree is simply an 'all the evidence' super-tree which at the very least tells us that overlapping genes have been around as long as some of the most ancestral clades in the prokaryotic group.

2. with respect to Figure 8: please give a histogram, not a "longer than" plot. The former would give us a better impression of the strength of the signal and the amount of data supporting it.

*We have added a panel to figure *8*containing the histogram of the number of occurrences.*

3. with respect to the phylogeny: do you observe any conservation of overlaps: i.e. rather than counting them, do the same genes overlap in phylogenetically "close" species. I guess it goes beyond the analyses in this paper, but there have been a lot of analyses done on the rate at which gene order is "randomized" in Bacteria and Archaea, also with respect to gene order. Phylogenetic conservation is always a strong argument for selection.

*While a detailed study of all 58 genomes for conserved sequences would be beyond the scope of this project, the analysis of pairs of closely related genomes, such as in the study *[10]*does support conservation.*

4. For table 5: did you take the codon bias in E. coli into account?

Yes, the expected fractions are computed by using the codon bias observed in E. coli

5. Conclusion 2: Are in all cases of overlapping co-directional gene pairs both genes indeed in the same operon? Either rephrase, or examine the available operon data, e.g. for E. coli.

F is this the case?

6. page 21: what does population size have to do with the rate of replication? Do know that Archaea are quite a bit slower in their replication than e.g. E. coli, and also in the Bacteria the differences are huge with some Bacteria (e.g. plantomycetes) having lower replication rates than some eukaryotes like yeast.

The point here is simply that prokaryote effective population sizes tend to be large and that this will make selection more efficient.

### Reviewer's report 3

Han Liang, Department of Ecology and Evolution, University of Chicago, USA (Nominated by Laura Landweber, Department of Ecology and Evolution, Princeton University

1. The study by Lillo and Krakauer represents a very comprehensive analysis of overlapping genes in prokaryotes. In particular, the carefully designed statistical analyses on the length and strand of overlapping sequences provides important insights into how different selective forces (i.e. genome minimization and co-regulation efficiency) shaped the evolution of overlapping genes. Overall, I think this is a valuable study that advances our understanding about the evolution of prokaryote genomes.

2. The authors presented the observation that no codirectional (123:123) phase was found as one of major results. Is it due to the bias in our current genome annotation? When a shorter ORF is embedded in a longer ORF with the same reading frame, only the longer one is reported. I also noticed that the embedded genes were not included in the dataset from the beginning. Thus, by definition, the codirectional (123:123) phase was excluded.

This is correct. We chose to study only non-embeded overlaps in order to arrive at a better understanding of differential patterns of phase usage.

3. The study specifically tested the stop codon mutation mechanism, where a mutation in a stop codon leads to read-through, thereby making two genes overlapped. But there is another alternative mechanism: a novel start codon can be created by mutations at the upstream of the second gene, leading to overlapping sequences. Discussion or further analysis on this aspect would be very helpful. The thing is that mutations occur without knowledge of translation orientation, and only their effects are evaluated by selection.

We have wrestled with this problem from the very start of this project. In one earlier version of the manuscript we had a section dealing explicitly with predictions derived from a model of ribosomal frame-shifting. This was eventually removed as there are numerous different motifs that are able to promote a ribosomal skip, and we found that we were unable to calculate the null expectation for overlapping genes under this mechanisms without a full look-up table of these sequences. The logic that you describe we share and this process is often observed in the process of differential gene translation in RNA viruses such as HIV. See section on stochastic origination

4. It is well known that there is a strong bias on stop codon usage and flanking nucleotide composition in most genomes. The comparison on stop codon usage (also flanking nucleotide bias) between overlapping and normal genes may generate some interesting results.

This is an interesting observation, and we have analyzed patterns of stop codon usage in both overlapping and non-overlapping genes. There are some differences, but we are not able to provide an explanation in this paper.
